# Influence of moderate-to-high intensity physical activity on depression levels: a study based on a health survey of Chinese university students

**DOI:** 10.1186/s12889-024-18433-w

**Published:** 2024-04-12

**Authors:** Bo Li, Wei Jiang, Shan-shan Han, Yu-peng Ye, Ya-xing Li, Hu Lou, Jun-yong Zhang

**Affiliations:** 1 Institute of Sports Science, Nantong University, 226019 Nantong, China; 2 School of Physical Education, Henan University of Economics and Law, 450046 Zhengzhou, China; 3 School of Physical Education, Jing-gang-shan University, 343009 Ji’an, China; 4 Physical Education College, Shangqiu University, 476000 Shangqiu, China

**Keywords:** College students, Physical inactivity, Mental health, Depression, Health promotion

## Abstract

**Objective:**

The study aims to examine how moderate-to-vigorous physical activity (MVPA) affects the severity of depression symptoms among Chinese college students. Additionally, it seeks to analyze the mediating mechanisms involving self-rated health and general self-efficacy.

**Methods:**

The study utilized data from the 2023 Chinese College Health Tracking Survey and employed multiple linear regression and structural equation modeling techniques to investigate the impacts of MVPA on depression levels and its underlying mediating mechanisms among college students. The primary cohort comprised 49,717 enrolled college students from 106 universities in China.

**Results:**

A total of 41,620 valid questionnaires were collected (response rate: 83.7%), with females accounting for 58.6%. In the past month, approximately 30.2% of college students engaged in MVPA. Self-rated health (B = − 0.282, *P* < 0.001) and general self-efficacy (B = − 0.133, *P* < 0.001) significantly influenced college students’ depression scores. Even after controlling for other variables, participating in MVPA remained significantly associated with reduced depression scores (B = − 0.062, *P* = 0.002). The results of the structural equation model showed that MVPA not only directly decreased college students’ depression scores but also indirectly reduced the likelihood of depression occurrence by improving their physical health status and general self-efficacy.

**Conclusion:**

The lack of physical activity among Chinese college students is evident. Engaging in MVPA can reduce the likelihood of depression among college students. MVPA achieves this reduction by enhancing college students’ general self-efficacy and improving their physical health. The factors influencing depression levels among college students are multifaceted. For future interventions targeting college students’ mental health, comprehensive approaches that incorporate behavioral and psychological factors should be emphasized.

## Background

Depression is a pervasive mood disorder. According to the World Health Organization, an estimated 350 million people worldwide grapple with this condition [1]. Symptoms of depression encompass a spectrum including sadness, loss of interest, hopelessness, tension, anxiety, and at times, even suicidal ideation [2, 3]. College students constitute a distinctive cohort within society. Given their pivotal role in social development and the critical stage they occupy for shaping values and personality, the mental health status of these individuals should not be overlooked.

The global incidence of mental health disorders, particularly depression, has been steadily increasing [4, 5]. College students undergo the transition from adolescence to adulthood. Owing to the influence of COVID-19 containment measures and their own negative thought patterns, the prevalence of depression among college students in China has noticeably escalated in recent years [6–8]. Depression heightens the risk of cardiovascular disease and overall mortality. It greatly contributes to disability-adjusted life years and the global burden of disease [9]. Recent research encompassing over 1.4 million individuals indicates that receiving a diagnosis of depression at any age is linked to a one to twofold increase in the risk of developing dementia [9]. Therefore, intervention measures aimed at addressing risk factors for depression must be implemented to enhance the mental health of college students.

Previous research has summarized various theoretical hypotheses regarding the mechanisms of how moderate-to-vigorous physical activity (MVPA) influences depression [10–12]. These hypotheses span cognitive–behavioral perspectives, encompassing psychological mechanisms, including cognitive-behavioral [13], social interaction [11], and distraction hypotheses [14]. Additionally, they involve physiological perspectives, such as the cardiovascular [15], amine [16], and endorphin hypotheses [17, 18]. This study employs the established theoretical framework found in existing literature, exploring psychological and physiological mechanisms that link MVPA with depression. The objective is to examine the influence of engaging in MVPA on depression levels among college students while establishing the mediating pathways through which MVPA affects these levels of depression. According to the conclusions of previous studies, MVPA has a positive effect on the mental health of college students [19–21]. Building upon this premise, the study posits Hypothesis 1: Engaging in MVPA can lower the likelihood of depression occurrence among college students.

The cognitive–behavioral hypothesis proposes that the antidepressant effects of MVPA (e.g., competitive sports) occurs by instigating positive cognitive and behavioral alterations in individuals. Bandura’s theory of self-efficacy, introduced in 1977 [22], posits that accomplishing challenging tasks can evoke positive psychological changes and boost self-efficacy [23]. For college students, adhering to a routine of regular and high-intensity physical exercise is frequently perceived as a challenging endeavor. Establishing a consistent exercise routine can bolster their sense of self-efficacy, potentially alleviating feelings of depression [24]. Therefore, this study proposes Hypothesis 2: MVPA diminishes depression levels in college students by augmenting their sense of self-efficacy.

Within the realm of relevant theoretical hypotheses concerning physiological mechanisms, the cardiovascular hypothesis posits that the antidepressant effects of exercise may be linked to cardiovascular health. The amine hypothesis and endorphin hypothesis suggest that the release of amine metabolites or endorphins during exercise may exert antidepressant effects [21]. Consistently engaging in MVPA as a lifestyle choice directly contributes to enhancing various physiological functions, including cardiovascular health, for college students. This approach helps them sustain a relatively favorable physical health status, consequently reducing anxiety and negative emotions triggered by employment, social pressure, academic challenges, and other stressors, thereby promoting their mental well-being. Therefore, this study proposes Hypothesis 3: MVPA diminishes depression levels in college students by enhancing their physical health.

## Methods

This study is a quantitative, observational research project aimed at exploring the relationship between participation in moderate-to-vigorous physical activity (MVPA) and depressive symptoms among university students, and to uncover potential mediating pathways. To achieve this, we compiled a sample of students from various universities and assessed their MVPA involvement, depressive symptoms, and a range of potential mediating variables. Data were collected through an online survey consisting of carefully selected and validated scales and questionnaires to ensure precise measurements of the constructs within our study. Participants were asked to report the frequency and duration of their weekly MVPA involvement, and depressive symptoms were evaluated using recognized mental health scales. In the data analysis phase, we began with descriptive statistical analysis and preliminary correlational analysis between variables. Subsequently, we employed Structural Equation Modeling (SEM) to assess the direct and indirect relationships between MVPA participation and depressive symptoms.

### Participants

The study was conducted using epidemiological survey methods, involving 49,717 college students from 106 universities across 31 provinces, autonomous regions, and municipalities in China. Out of these, 41,620 valid questionnaires were collected. Among the respondents, 24,408 were female, constituting 58.6% of the total sample. The survey targeted students enrolled in regular higher education institutions on the Chinese mainland, following the “National List of Regular Higher Education Institutions (as of June 15, 2023)” provided by the Ministry of Education. This survey encompassed students pursuing associate and bachelor’s degrees but excluded graduate students (master’s and doctoral programs). Verbal consent was obtained from every participant before data collection, and the right to withdraw from the study at any point was guaranteed. Confidentiality was protected for all participants via the non-disclosure of identity. The survey employed a stratified, cluster, and staged sampling method, with the following specific sampling steps.

### Determination of sampling sites

To ensure the representativeness of the surveyed population, three sampling sites were evenly allocated within each province, autonomous region, or municipality. These sites were selected from cities at the provincial or regional level. Among these, the provincial capital city was designated as a “Level One” sampling site. Because the provincial capital cities often represent the most economically developed cities in a province. The other two cities were chosen based on principles that accounted for the geographical diversity of the province or region and represented varying levels of socioeconomic development: one city representing a moderately developed socioeconomic level (“Level Two”) and another reflecting a relatively lower socioeconomic development level (“Level Three”). Sampling in the directly-administered municipalities did not strictly adhere to the principles but emphasized random cluster sampling. However, the sampling methodology still aimed to balance the number of samples across the three designated sampling sites.

### Determination of sampling units

The selection of sampling units primarily considered three factors: Firstly, the affiliated higher education institutions needed to be officially recognized and registered by the Ministry of Education, encompassing vocational and technical colleges. Secondly, the units had to fulfill sampling criteria concerning factors like age range, student population, and grade distribution. Thirdly, each sampling unit needed an assigned person responsible for distributing questionnaires and displaying a willingness to participate in long-term monitoring. Additionally, the college students at the chosen sampling units had already resumed on-campus activities for the fall semester.

### Grouping and sample size

The population was initially categorized by gender (male and female), and further subdivided into eight groups based on grade distinctions. Each category (e.g., male first-year university students) maintained a minimum sample size of 45 students. For each province, autonomous region, or municipality, the total sample size reached 1,080 students, aiming for an estimated nationwide response of 33,480 completed questionnaires (excluding Hong Kong, Macau, and Taiwan). The survey, conducted via the Questionnaire Star software, employed an electronic questionnaire uniformly administered during two teaching weeks in October 2023 (from October 9th to 22nd), resulting in the collection of 49,717 questionnaires.

## Measurement

The survey questionnaire comprises sections on sociodemographic details, physical activity (PA), depressive symptoms, general self-efficacy, and self-rated health. On average, it takes approximately 5 min and 56 s to complete the entire questionnaire. Participants were asked to answer a total of 30 questions in 6 areas. Ensuring the quality of the questionnaire analysis, the selected measurement scales have undergone testing for psychometric properties specifically among Chinese university students, and they are accompanied by Chinese norms. The measurement primarily emphasizes the height and weight status of the students. The key measurement and analysis indicators are outlined as follows.

The sociodemographic information encompasses gender, grade, age, school attended, and the school’s location (with the provision of a postal code). The school’s location is categorized into three regions: “Eastern,” “Central,” and “Western,” based on China’s geographical division. The Eastern region comprises Beijing, Tianjin, Hebei, Liaoning, Shanghai, Jiangsu, Zhejiang, Fujian, Shandong, Guangdong, and Hainan provinces; the Central region includes Shanxi, Jilin, Heilongjiang, Anhui, Jiangxi, Henan, Hubei, and Hunan provinces; the Western region encompasses Sichuan, Chongqing, Guizhou, Yunnan, Shaanxi, Gansu, Qinghai, Ningxia, Xinjiang, Guangxi, and Inner Mongolia [25].

The International PA Questionnaire-Short Form (IPAQ) is utilized to evaluate the PA levels of university students [26]. Following scoring rules, the PA levels are categorized into “Vigorous Intensity PA (VPA),” “Moderate Intensity PA (MPA),” and “Light Intensity PA (LPA)”. VPA and MPA are combined as MVPA. The questionnaire has demonstrated remarkable reliability and validity in the Chinese population [27, 28]. In a study conducted by Qu, the agreement rate between the short form and the goal PA activity measured by a PA record reached over 70% among university students, and the correlation coefficient (r) of different PA categories between two tests ranged from 0.626 to 0.887, suggesting that the test–retest reliability and validity of IPAQ in university students are higher than or equal to those of similar questionnaires [29]. In a study performed by Xu, the correlation coefficient between the questionnaire and the PA measured by an accelerometer in university students was 0.543, indicating a good overall effect in measuring PA levels [30].

Depression level was assessed using the Chinese version of the Center for Epidemiological Studies Depression Scale (CES-D), following the methods described in the “Psychological Health Blue Book: Chinese National Mental Health Development Report (2019–2020)“ [31]. CES-D is widely used internationally for screening depressive symptoms in the general population. Respondents were required to rate the frequency of symptoms in past weeks on a scale of 0 to 3. A cutoff score of 10 and a high-risk cutoff score of 17 were used [32]. He et al. (2013) tested the reliability and validity of CES-D on a nationally representative sample of 30,801 individuals and found that the internal consistency reliability of CES-D ranged from 0.85 to 0.88, with a test–retest reliability of 0.49 (*P* < 0.001). All items were positively correlated with the total score, indicating good reliability and validity, thereby making CES-D suitable for measuring depressive levels across different age groups. In our study, the internal consistency reliability of CES-D, measured by Cronbach’s alpha, was 0.822 (positive items) and 0.859 (reverse items). The test–retest reliability coefficient measured by Pearson’s correlation coefficient was 0.619.

General perceived self-efficacy was measured using the General Perceived Self-efficacy Scale (GSES) among university students. The GSES was developed by Schwarzer and translated and revised into Chinese by Wang et al [33, 34], with its reliability and validity analyzed. The results showed that GSES suggested good reliability, with a Cronbach’s alpha coefficient of 0.87 and a test-retest reliability coefficient of 0.83 (*P* < 0.001). The split-half reliability coefficient was 0.82 (*n* = 401, *p* < 0.001).

Self-rated health was measured using the Medical Outcomes Study (MOS) 36-item Short Form Health Survey (SF-36) to assess the self-perceived health status of university students. The SF-36 is a widely used generic instrument developed by the MOS for measuring health-related quality of life. The Chinese version was translated by the Department of Social Medicine at Zhejiang University School of Medicine in 1991. In this study, the “general health (GH)” domain of the SF-36 was selected for analysis. The SF-36 showed good reliability and validity in Chinese university students, with a split-half reliability coefficient of 0.92, a Cronbach’s alpha coefficient of 0.88, a success rate of 100% for convergent validity, and a success rate of 98.4% for discriminant validity. It has demonstrated good reliability and validity in measuring the health status of university students and can be used in practice [35].

BMI was measured using the Yan Yan brand medical weight scale (model: RGZ-160) to assess the height and weight of university students, which is classified based on the standards outlined in the “*National College Student Physical Health Standards.*” The classification criteria for BMI of college students are presented in Table 1.

**Table 1:** Body mass index (BMI) scoring sheet (Unit: kg/m2)

Categories	Male	Female
Normal	17.9 ~ 23.9	17.2 ~ 23.9
Underweight	≤ 17.8	≤ 17.1
Overweight	24.0 ~ 27.9	24.0 ~ 27.9
Obesity	≥ 28.0	≥ 28.0

## Statistical analysis

Data processing was performed using SPSS 25.0 and Excel software. First, data preprocessing, involving the selection of valid questionnaires according to the criteria for effective data filtering, was conducted. Next, chi-square tests were used to analyze the differences in MVPA based on demographic variables and BMI classification. The effect size in the analysis of differences was measured using Cramer’s V coefficient (V coefficient) [36]. Independent sample t-tests and one-way ANOVA were employed to examine the differences in CES-D, GSES, and SF-6 scores based on demographic and BMI categories.

Multiple linear regression modeling was utilized to analyze the influencing factors of depressive levels among university students, verifying whether participation in MVPA affects their depressive symptoms. During the statistical analysis, the dependent variable depression was statistically analyzed using the raw score of the CES-D. Categorical variables such as gender, grade, school region, and ethnicity are set as dumb variables. The independent variables self-rated health, BMI, and general self-efficacy were continuous.

Additionally, a structural equation model (SEM) was applied to analyze the pathways through which participation in MVPA affects depressive symptoms among university students. To implement the structural equation model, we utilized SPSSAU software. Due to the inclusion of most categorical variables as independent variables in the SEM, this study utilized weighted least squares mean and variance-adjusted estimation modeling, with the Probit function as the linking function.

## Results

### The descriptive characteristics of the study participants

Differential analysis was conducted to examine the levels of depressive symptoms among university students with unique characteristics (Table 2). The results indicated variations in depressive symptoms among students from different regions, with students from the eastern region exhibiting the highest CES-D scores (9.109 ± 4.697). Gender differences were observed in the participation of university students in MVPA over the past month (V = 0.311), specifically, the proportion of females (18.3%) engaging in MVPA was lower than that of males (47.3%). Overall, university students predominantly engaged in light-intensity PA (LPA), accounting for 69.8% of the total. Among different BMI classifications, overweight students (33.4%) reported the highest proportion of participation in MVPA (V = 0.161).Among different ethnic groups, the participation rate of the Han population in MVPA (29.6%) is lower than that of the ethnic minorities (38.3%).

**Table 2:** The descriptive characteristics of the study participants

Variable	Number	%	Depression			Participation in MVPA		General self-efficacy			Self-rated health		
			M	SD	Statistical value	%	Statistical value	M	SD	Statistical value	M	SD	Statistical value
Overall	41,620	100	9.022	4.601		30.2		2.78	0.54		62.464	19.162	
**Gender**													
Male	17,212	41.4	8.991	4.575	t=-1.158 *P* = 0.247	47.3	x2 = 4021.004 *P* < 0.001 **V = 0.311**	2.776	0.539	t=-1.367 *P* = 0.172	62.504	19.118	t=-1.158 *P* = 0.247
Female	24,408	58.6	9.044	4.62		18.3		2.783	0.541		62.436	19.193	
**Grade**													
Freshman	23,878	57.4	9.04	4.588	F = 0.566 *P* = 0.638	31.2	x2 = 63.342 *P* < 0.001 V = 0.039	2.777	0.537	F = 10.762 *P* < 0.001	62.201	19.143	F = 9.536 *P* < 0.001
Sophomore	10,605	25.5	9.022	4.601		30.3		2.77	0.546		62.36	19.144	
Junior	5479	13.2	8.952	4.69		25.8		2.818	0.547		63.712	19.291	
Senior	1658	4	8.995	4.497		31.2		2.772	0.53		62.8	18.972	
**Area**													
Eastern	14,657	35.2	9.109	4.697	F = 5.055 *P* = 0.001	29.6	x2 = 118.938 *P* < 0.001 V = 0.053	2.786	0.545	F = 14.027 *P* < 0.001	62.518	19.137	F = 1.410 *P* = 0.224
Westward	10,300	24.7	8.923	4.472		34.4		2.756	0.531		62.676	19.182	
Central	16,663	40	9.007	4.594		28.2		2.79	0.542		62.286	19.171	
**Ethnicity**													
Han	38,360	92.2	9.016	4.596	t=-0.095 *P* = 0.335	29.6	x2 = 108.157 *P* < 0.001 V = 0.051	2.782	0.541	t = 2.308 *P* = 0.042	62.44	19.148	t=-0.892 *P* = 0.372
Minority	3260	7.8	9.097	4.66		38.3		2.762	0.537		62.752	19.33	
**BMI**													
Underweight	4608	11.1	9.018	4.569	F = 0.501 *P* = 0.682	22.7	x2 = 555.629 *P* < 0.001 **V = 0.161**	2.789	0.542	F = 0.991 *P* = 0.392	61.687	19.156	F = 3.652 *P* = 0.012
Normal	24,976	60	9.035	4.59		30.8		2.777	0.541		62.468	19.155	
Overweight	5563	13.4	8.954	4.612		33.4		2.785	0.539		62.644	18.968	
Obesity	6433	15.5	9.039	4.661		30.9		2.783	0.541		62.87	19.366	

With regard to general self-efficacy measurement among university students, variations were observed among different academic years (F = 10.762, *P* < 0.001), with third-year students (2.818 ± 0.547) exhibiting the highest scores in general self-efficacy. Additionally, students from the central region (2.790 ± 0.542) reported the highest scores in general self-efficacy. Differences were found in self-rated health scores among university students of different academic years (F = 9.536, *P* < 0.001), with third-year students (63.712 ± 19.291) having the highest self-rated health scores.

### Influence of MVPA on university students’ depressive symptoms

Reply to Q1: This (+) is a clerical error, please delete it directly. Thank you for your careful and meticulous work. Please note: The above is the author's response to Q1 and is not the content of the article.

This study conducted multiple linear regression analyses to further analyze the relationship between university students’ participation in MVPA and depressive symptoms. The CES-D score was used as the dependent variable, while participating in MVPA over the past month was spent as the independent variable. Four dimensions of control variables, including demographic characteristics, physical health status, and general self-efficacy, were included in the analysis (Table 3). Model 1 only included demographic characteristics, Model 2 simultaneously incorporated demographic characteristics and self-rated health factors, Model 3 added BMI status on top of Model 2, Model 4 included general self-efficacy, and Model 5 introduced the key independent variables, MVPA, in addition to all the control variables.

**Table 3:** Results of multivariate linear regression analysis on factors influencing the level of depressive symptoms among college students

Variable	Model 1			Model 2			Model 3			Model 4			Model 5		
	B	SE	P	B	SE	P	B	SE	P	B	SE	P	B	SE	P
Gender_Male	-0.010	0.010	0.332	-0.008	0.010	0.420	-0.009	0.010	0.375	-0.007	0.010	0.457	-0.004	0.010	0.725
Grade_Sophomore	-0.006	0.012	0.605	-0.003	0.011	0.759	-0.003	0.011	0.758	-0.001	0.011	0.914	-0.001	0.011	0.928
Grade_Junior	0.024	0.015	0.117	0.001	0.015	0.963	0.001	0.015	0.967	-0.005	0.015	0.719	-0.005	0.015	0.711
Grade_Senior	-0.016	0.025	0.520	-0.006	0.024	0.794	-0.006	0.024	0.794	-0.003	0.024	0.900	-0.003	0.024	0.905
Area_Westward	-0.043	0.013	0.001	-0.039	0.013	0.002	-0.039	0.013	0.002	-0.032	0.012	0.009	-0.032	0.012	0.010
Area_Central	-0.019	0.011	0.104	-0.024	0.011	0.032	-0.024	0.011	0.031	-0.025	0.011	0.025	-0.025	0.011	0.022
Ethnicity_Han	-0.022	0.019	0.229	-0.026	0.018	0.144	-0.026	0.018	0.145	-0.029	0.018	0.102	-0.030	0.018	0.093
Self-rated health.				-0.282	0.005	< 0.001	-0.282	0.005	< 0.001	-0.314	0.005	< 0.001	-0.314	0.005	< 0.001
BMI							0.002	0.005	0.609	0.003	0.005	0.583	0.002	0.005	0.604
General self-efficacy										-0.133	0.005	< 0.001	-0.123	0.005	< 0.001
Participation in MVPA													-0.062	0.011	0.002
Constant term	0.048	0.020	0.015	0.047	0.019	0.013	0.048	0.019	0.012	0.049	0.019	0.010	0.052	0.019	0.007
Adjusted R-squared	0.060			0.080			0.081			0.097			0.108		

The results of this study revealed a significant negative correlation between MVPA and university students’ depressive symptoms, thereby confirming Hypothesis 1. Model 5 examined the effects of all control and independent variables on depressive symptoms among university students. Compared with Model 4, the inclusion of the MVPA variable in Model 5 improved the overall explanatory power of the model (adjusted R^2^ = 0.108). Furthermore, the results of Model 5 demonstrated that, even after controlling for demographic characteristics, physical health status, general self-efficacy, and other variables, participation in MVPA still exerted a substantial influence on reducing depressive symptoms among university students. On average, the level of depressive symptoms decreased by 0.062 points among university students who engaged in MVPA, compared with those who did not participate in MVPA, indicating that overall, participating in MVPA has a positive effect in reducing the occurrence of depressive symptoms among university students.

Self-rated health and general self-efficacy may mediate the effects of MVPA on reducing depressive symptoms. The model results indicated that self-rated health status substantially influenced the occurrence of depressive symptoms among university students, with students who perceived their own health as worse being more susceptible to experiencing depressive symptoms. General self-efficacy also displayed a noteworthy effect on university students’ depressive symptoms, with higher levels of general self-efficacy associated with a considerable reduction in depressive symptoms. These findings support the hypotheses of this study, suggesting that university students’ engagement in MVPA may potentially reduce depressive symptoms from the perspectives of self-rated health and general self-efficacy.

### Pathway mechanism of MVPA’s influence on university students’ depressive symptoms

**Figure 1:**
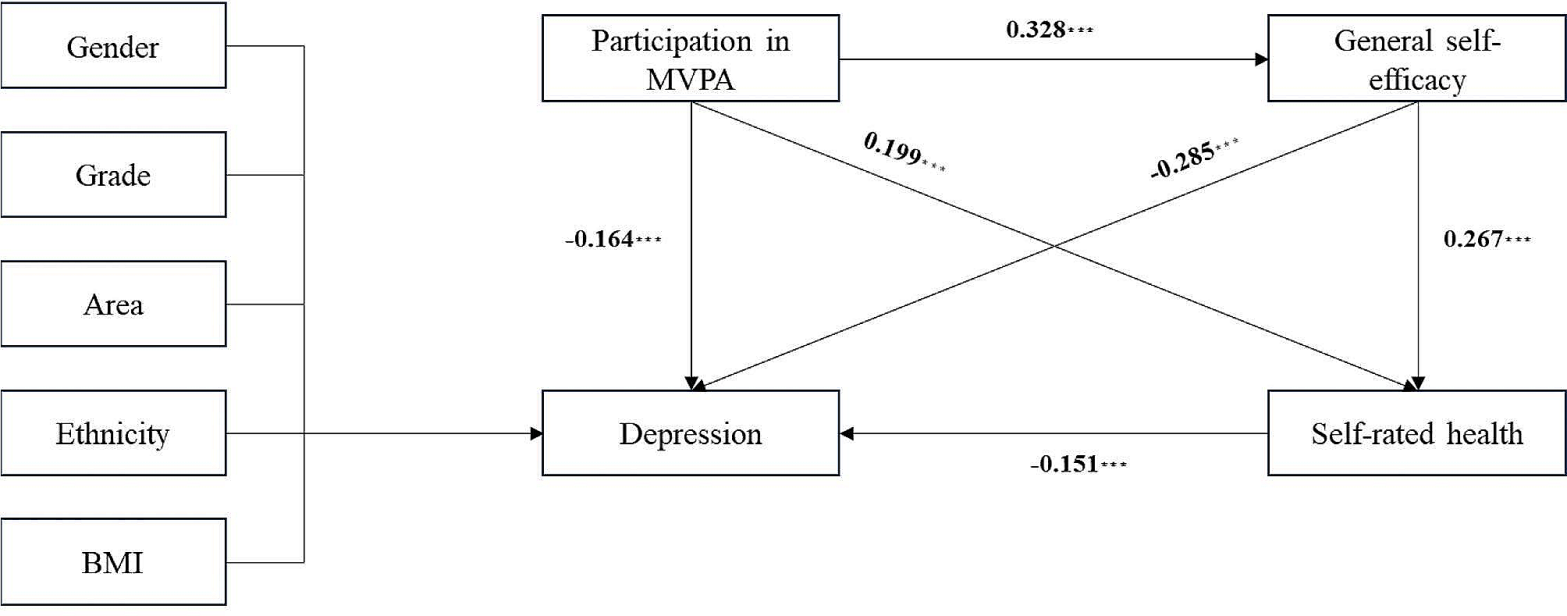
Structural equation model of the impact of participation in MVPA on depression levels among college students

To further investigate the pathway mechanism through which participation in MVPA influences university students’ depressive symptoms, this study employed SEM analysis (Fig. 1). The model fit indices were as follows: x^2^/df = 3.869 < 0.500, RMSEA = 0.091 < 0.100, NFI = 0.936 > 0.900, CFI = 0.901 > 0.900, and AGFI = 0.896 > 0.800, indicating a satisfactory fit of SEM.

The model showed that the direct effect of college students’ participation in MVPA on depression was − 0.164, and *P* < 0.001, which may mean that the higher the degree of MVPA participation, the lower the degree of depressive symptoms among college students. MVPA affected self-rated health (0.285, *P* < 0.001) and then depression (-0.151, *P* < 0.001), and the total effect of this indirect pathway was − 0.043, and each pathway was significant. MVPA also affected depression (0.199, *P* < 0.001) through general self-efficacy (0.328, *P* < 0.001), and the total effect of this indirect pathway was 0.065, again with each pathway being significant.

The results of the model demonstrated that general self-efficacy and self-rated health factors partially mediated the relationship between MVPA and depressive symptoms, with a relatively high proportion of indirect effects. Participation in MVPA enhanced university students’ general self-efficacy, which in turn reduced the likelihood of experiencing depressive symptoms, thus validating Hypothesis 2. Additionally, the results of the SEM also indicated that MVPA indirectly influenced the depressive symptoms of university students through its impact on their self-rated health, thus validating Hypothesis 3. MVPA exerted a direct positive effect on the psychological well-being of university students by maintaining their physical health. Furthermore, in view of the inseparable link between physical and mental health, it helped create a more positive psychological state, thereby reducing the likelihood of the occurrence of depressive symptoms.

## Discussion

This study adopts an epidemiological survey paradigm to investigate the correlation between university students’ engagement in MVPA and the severity of depressive symptoms. The research introduces variables such as physical health and self-efficacy to explore the pathways and mechanisms through which MVPA may influence depression. These findings hold substantial practical implications, particularly considering the prevalent mental health challenges among Chinese university students.

He study findings reveal that the majority of university students’ PA (PA) levels are categorized as low-level PA, constituting 69.8% of the sample. Moreover, male students exhibit higher engagement in MVPA compared to female students. Globally, a significant trend of decreasing PA levels is observed across various populations due to changes in nutrition, transportation, and lifestyle [37]. This phenomenon is closely associated with the decline in physical fitness and health, as evidenced by the eighth national survey on students’ physical fitness and health released by the Chinese Ministry of Education in 2021. This survey showed that the overall rate of students meeting physical fitness standards has not improved, and the failure rate stands at a high level of 30% [38]. In terms of gender differences, this survey aligns with previous research findings that male students engage in more physical exercise than female students [39, 40]. Studies have found that during the transition from adolescence to adulthood, male and female students show an increase in MVPA before the age of 12, followed by a continuous decline, which may be related to the rapid physiological and psychological development during adolescence [41]. Physical inactivity is prevalent across various populations globally, highlighting the importance of promoting PA by adhering to the principles of “ more movement is better, moderation is key, and persistence is crucial,” as outlined in the “Physical Activity Guidelines for the Chinese Population (2021).” Despite higher scores in self-efficacy and health self-assessments amongst third-year college students, our study identifies a notable shortfall in their participation in moderate to vigorous physical activity (MVPA). This paradox underscores not only the limitations inherent to self-assessment methodologies but also a propensity among young adults to overestimate their own health and activity levels. The intersection of developmental, cognitive, and social factors at this stage further exacerbates this tendency, leading to a significant discordance between self-perception and actual behavior. Standard statistical tools, such as mean values and standard deviations, were employed to quantify participant performance, yet may fall short in capturing the full complexity of physical activity and the intricacies of its cognitive perception. This reflection prompts an essential consideration for deeper investigation into the dynamics underpinning self-reported health and behavior, paving the way for more nuanced analyses and informed interventions.

Socioeconomic status may be a factor that influence the level of depression among university students. China is a country with considerable regional disparities, with varying levels of economic development and resource allocation across different areas. In this study, the geographical location of universities where students are enrolled is divided into three categories, namely, eastern, central, and western regions. From the perspective of socioeconomic development, the eastern region is superior to the western and central regions. The results of this study show differences in depression levels among students from different geographical locations. Students from the eastern region have the highest CES-D scores (9.109 ± 4.697), indicating a higher level of depression compared with the western and central regions. This finding differs drastically from previous research [42, 43], because most previous studies suggested that lower socioeconomic status may lead to increased psychological stress, lack of resources, and insufficient social support, thereby increasing the risk of depression. However, the results of this study suggest the opposite, possibly because the eastern region of China has a higher level of socioeconomic development. Additionally, many first-year students who come from different regions of China to their university’s location were surveyed. The differences between their hometown and the university’s location may contribute to depressive symptoms. Of course, socioeconomic status includes factors, such as income, education level, and occupation [44]. Further comprehensive empirical research is needed to explore the effects of this indicator on depression among university students.

Differences are observed in self-reported MVPA among university students in different BMI categories, with overweight students (33.4%) reporting the highest proportion of MVPA participation (V = 0.161). These findings differ from previous research, such as Caballero et al., which suggested that overweight and obese students are the main group with insufficient PA [45], and Rachan’s study found a high and significant positive correlation between weight status and BMI among university students [46]. However, the results of this study indicate that more overweight and obese students perceive themselves as participating in MVPA compared with normal-weight students, which may be related to the measurement attribute of IPAQ. In the IPAQ, participants are required to self-report the intensity levels of each type of activity, including light, moderate, and vigorous, based on their perceived levels of physical fatigue, breathlessness, or sweating induced by the activity [26]. Compared with normal-weight students, overweight and obese students may experience stronger feelings of fatigue during the same type of PA, leading to an increase in self-reported MVPA levels. This phenomenon highlights a limitation of self-reporting in measuring PA [47, 48].

Participation in MVPA can reduce the risk of depression among university students. Regression analysis shows that factors, such as physical health and self-efficacy, greatly influence students’ depression scores. After controlling other variables, participation in MVPA continues to have a substantial effect in reducing students’ depression scores, contributing to an overall improvement in the model’s explanatory power. This finding validates the positive psychological effects of college students engaging in MVPA. Currently, various hypotheses regarding exercise’s promotion of mental health are available [49, 50]. In the context of this study, the “enhanced self-esteem and self-confidence hypothesis” and the “neurobiological hypothesis” can be used to explain the findings.

The enhanced self-esteem and self-confidence hypothesis posit that engaging in sports or exercise activities can bolster individuals’ self-evaluation, self-esteem, and self-confidence, potentially resulting in positive effects on their mental health [51–53]. This study employed a specific hypothesis to examine the second hypothesis, which suggested that engaging in Moderate to Vigorous Physical Activity (MVPA) could reduce depressive symptoms in university students through the fortification of their self-efficacy. To delve into the potential intermediary effects that general self-efficacy and physical health status may hold in the relationship between MVPA and depressive symptoms, a mediator hypothesis model grounded in self-efficacy theory and the physiological factor hypothesis was developed. The empirical evidence revealed a significant association: as self-efficacy increased, depressive scores among university students decreased. The findings underscore the pivotal role of MVPA in diminishing depressive symptoms by bolstering self-efficacy. The analysis suggests that engaging in MVPA can help cultivate self-efficacy among university students, which refers to their confidence in effectively completing tasks and coping with challenges. By achieving goals during MVPA, students can experience their own abilities and strengths, thereby reinforcing positive self-perception and ultimately reducing depressive symptoms [54].

The neurobiological hypothesis suggests that exercise can alter neurotransmitters and brain neurodevelopment, leading to an increased release of chemicals such as dopamine, serotonin, and endorphins. These chemicals play crucial roles in emotion regulation and pleasure, and their release during exercise can enhance psychological well-being [51, 55, 56]. This hypothesis supports Hypothesis 3 of the current study, proposing that engaging in MVPA can diminish depressive symptoms among university students by enhancing their physical health status.

This study sought to explore the mechanisms through which MVPA affects the mental health of university students by examining the mediating roles of physical health and self-efficacy. The findings validated that MVPA not only contributes to better physical well-being but also bolsters self-efficacy, consequently enhancing the psychological well-being of university students. Moreover, the study pinpointed diverse factors that influence the engagement of various student groups in MVPA, indicating the necessity for tailored policies and targeted resource allocation to deliver personalized sports-related public services to university students.

Limitations of this study involve relying solely on indicators of MVPA to gauge PA levels among university students, potentially lacking comprehensive information. Additionally, using retrospective self-report questionnaires for assessing PA introduces the risk of measurement bias. Furthermore, further research is needed to elaborate on the pathways and mechanisms that influence the connection between MVPA and depressive symptoms. Future studies could benefit from using other focused questionnaire designs or referencing similar experimental studies conducted by international scholars to explore the relationship and underlying mechanisms between MVPA and depressive symptoms among university students, utilizing experimental and control groups.

## Conclusions

The phenomenon of insufficient PA among Chinese university students is evident. Participating in MVPA has been linked to a decreased likelihood of depression among these students. MVPA appears to alleviate depressive symptoms by bolstering students’ self-efficacy and improving their physical health. The factors influencing the level of depressive symptoms among university students are multifaceted. Future interventions aimed at enhancing the psychological well-being of university students should prioritize integrated approaches that encompass behavioral and psychological components. Since our study utilizes a cross-sectional design, we can only observe an association between MVPA and depressive symptoms, but cannot draw causal inferences. Moreover, cross-sectional studies typically provide a snapshot of data at a single point in time, failing to capture how variables change and develop over time. Therefore, our study does not reveal how MVPA influences depressive symptoms over time, or how this effect might vary as time progresses. To address this issue, we need to carry out longitudinal study designs to track and evaluate the long-term dynamic relationship between MVPA and depressive symptoms.

## Data Availability

The raw data supporting the conclusions of this article will be available from Bo Li (Wangqiulibo@163.com) on reasonable requests.

## Abbreviations

CES-D: Center for Epidemiological Studies Depression Scale
COVID-19: Coronavirus disease 2019
IPAQ: International Physical Activity Questionnaire
LPA: Light intensity PA
MH: Mental health
MPA: Moderate intensity PA
MVPA: Moderate to vigorous intensity PA
PA: Physical activity
SF-36: Short Form Health Survey
VPA: Vigorous intensity PA
